# Is It Possible to Expand Oocyte Donors by Decreasing Number of Oocytes for Own Use? Insights From a Large Single-Center Study

**DOI:** 10.3389/fendo.2021.727339

**Published:** 2021-11-11

**Authors:** Zhiqin Bu, Jiaxin Zhang, Yile Zhang, Yingpu Sun

**Affiliations:** Reproductive Medical Center, First Affiliated Hospital of Zhengzhou University, Zhengzhou, China

**Keywords:** oocyte donation, cumulative live birth rate, supernumerary embryo, IVF, China

## Abstract

**Background:**

Currently, in China, only women undergoing *in vitro* fertilization/intracytoplasmic sperm injection (IVF/ICSI) cycles can donate oocytes to others, but at least 15 oocytes must be kept for their own treatment. Thus, the aim of this study was to determine whether oocyte donation compromises the cumulative live birth rate (CLBR) of donors and whether it is possible to expand oocyte donors’ crowd.

**Methods:**

This was a retrospective cohort study from August 2015 to July 2017 including a total of 2,144 patients, in which 830 IVF–embryo transfer (IVF-ET) patients were eligible for oocyte donation and 1,314 patients met all other oocyte donation criteria but had fewer oocytes retrieved (10–17 oocytes). All 830 patients were advised to donate approximately three to five oocytes to others and were eventually divided into two groups: the oocyte donation group (those who donated) and the control group (those who declined). The basic patient parameters and CLBR, as well as the number of supernumerary embryos after achieving live birth, were compared. These two factors were also compared in all patients (2,144) with oocyte ≥10.

**Results:**

In 830 IVF-ET patients who were eligible for oocyte donation, only the oocyte number was significantly different between two groups, and the donation group had more than the control group (25.49 ± 5.76 vs. 22.88 ± 5.11, respectively; p = 0.09). No significant differences were found between the two groups in other factors. The results indicate that the live birth rate in the donation group was higher than that in the control group (81.31% vs. 82.95%, p = 0.371), without significance. In addition, CLBR can still reach as high as 73% when the oocyte number for own use was 10. Supernumerary embryos also increased as the oocyte number increased in all patients (oocyte ≥10).

**Conclusions:**

Currently, oocyte donation did not compromise CLBR, and oocyte donation can decrease the waste of embryos. In addition, in patients with 10 oocytes retrieved, the CLBR was still good (73%). Thus, it is possible to expand oocyte donors if the number of oocyte kept for own use was decreased from 15 to 10 after enough communication with patients.

## Introduction

As the social, economic, and environmental factors changed rapidly, the Chinese government implemented two-child policy and even three-child policy in recent years, causing the number of women of advanced maternal age to grow quickly (1–4). The available evidence suggests that human fertility declines with advancing age; therefore, there is a steady increase in the demand for assisted reproductive treatment among older women (5). It is well known that advanced age is negatively associated with fertility, and reproductive failure could be mostly due to ovarian factors rather than uterine factors (6). Women who are unable to conceive using autologous oocytes could use donated oocytes to become pregnant. Oocyte sharing has been used for many years in some countries (7, 8). However, it has been estimated in China that 85%–95% of women who are in need of egg donation cannot receive treatment because they do not have their own gametes.

The pool of oocyte donors is restricted to sterile women who themselves undergo assisted reproduction cycles with autologous oocytes (9). Consequently, Chinese anovulation infertility women have been suffering from a severe shortage of egg donors, causing long waiting lists and limited choices. What bothered the donors was that the oocyte decrease would reduce the number of oocytes available to them for fertilization, which was likely to harm their own interests. A similar retrospective analysis was performed and demonstrated that there is no detrimental effect on the live birth rate (10). To the best of our knowledge, there have been no such Chinese reports concerning this question, and thus, we conducted a study to examine this issue in China.

## Materials and Methods

This retrospective study included women undergoing their first *in vitro* fertilization/intracytoplasmic sperm injection (IVF/ICSI) cycles between August 2015 and July 2017. All patients’ basic characteristics and IVF/ICSI treatment outcomes were recorded in the Clinical Reproductive Medicine Management System/Electronic Medical Record Cohort Database (CCRM/EMRCD) in Reproductive Medical Center, First Affiliated Hospital of Zhengzhou University. All patients included in the study were of reproductive age, and their oocyte reserve function was good. Therefore, they were applied with agonist long protocol routinely. The inclusion criteria for oocyte donation were as follows: age <35 years and number of oocyte retrieved ≥18. According to Documents No. 176 (2003) and No. 44 (2006) of the National Health Commission of the People’s Republic of China, the criteria for human oocyte donation screening are similar with the requirements of sperm; previous systemic diseases, exposure history to toxic or radioactive substances, sexually transmitted diseases, and genetic history are excluded. The exclusion criteria were as follows: preimplantation genetic testing (PGT); body mass index (BMI) ≥30 kg/m^2^; polycystic ovary syndrome (PCOS); infectious diseases; inherited disease; family history of inheritable disease; chronic disease; and reproductive system diseases, such as endometriosis, hydrosalpinx, or uterine malformation. This study has been approved by the Institutional Review Board (IRB) of the First Affiliated Hospital of Zhengzhou University.

### Oocyte Donation

Controlled ovarian stimulation was performed with gonadotropin-releasing hormone agonist (GnRH-a) pituitary downregulation protocols, which have been described previously (11).

On the day of oocyte retrieval, women eligible for oocyte donation were advised to donate three to five oocytes to others. The donated oocytes were fertilized with frozen sperm from husbands of oocyte recipients. Day 3 embryos will be frozen for at least 6 months to wait oocyte donors’ final results of infectious diseases test (hepatitis B, hepatitis C, syphilis, and HIV). The follow-up rate of each donation cycle was 100%, and each patient’s personal information was confidential (12).

### Date Analysis

**Figure 1:**
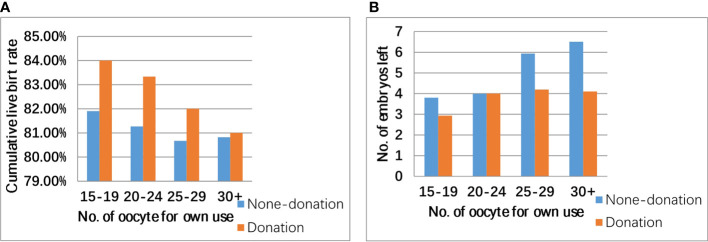
Cumulative live birth rate **(A)**, number of supernumerary embryo **(B)**, and number of oocytes retrieved in patients eligible for oocyte donation (oocyte ≥18).

First, the patients included were divided into two groups: the oocyte donation group (those who donated) and the control group (those who declined). Basic characteristics, such as age, BMI, baseline follicle-stimulating hormone (FSH), number of oocytes retrieved, and number of supernumerary embryos after live birth, are shown as the mean ± standard deviation (SD); and Student’s t-test was used to detect differences between the two groups. The implantation rate, live birth rate in first transfer, cumulative live birth rate (CLBR), and biochemical miscarriage rate were compared between the two groups using chi-square analysis. Cumulative rate, supernumerary embryos, and number of oocytes (oocyte ≥18) were compared between the oocyte donation and control groups in **Figure 1**. In addition, we also included those who met all other oocyte donation criteria but had fewer oocytes retrieved (10–17 oocytes). Their CLBR and number of supernumerary embryos after live birth are also illustrated in bar graphs.

All data were analyzed in SPSS (Statistical Package for the Social Sciences, SPSS Inc., Chicago, IL) 22.0. It was considered to be statistically significant when p < 0.05.

## Results

In total, data from 2,144 IVF-ET cycles performed in the Reproductive Center from August 2015 to July 2017 were included among 830 patients who met the conditions to donate oocytes. The two groups of patients’ basic characteristics are shown in **Table 1**. Only the oocyte number was significantly different between the two groups; the donation group had more oocytes than the control group (25.49 ± 5.76 vs. 22.88 ± 5.11, p = 0.009). There were no significant differences between the two groups in basic parameters, implantation rate, live birth rate in the first transfer, biochemical miscarriage rate, and embryos obtained. The oocyte number in the donation group was larger than that in the control group before donation, but the actual number of oocyte for own use was comparable (21.42 ± 5.37 vs. 22.88 ± 5.11, p = 0.098).

**Table 1:** Basic characteristics of two groups.

	Non-donation	Donation	p
Patient age (years)	27.72 ± 3.24	27.30 ± 3.31	0.657
BMI (kg/m^2^)	22.33 ± 2.90	22.15 ± 2.69	0.217
Baseline FSH (IU/L)	5.97 ± 1.46	5.65 ± 1.31	0.609
No. of oocyte retrieved	22.88 ± 5.11	25.49 ± 5.76	0.009
Actual No. of oocyte for own use	22.88 ± 5.11	21.42 ± 5.37	0.098
Implantation rate	55.00% (286/520)	56.25% (45/80)	0.834
Live birth rate in the first transfer	54.92% (385/701)	52.71% (68/129)	0.643
Cumulative live birth rate	81.31% (570/701)	82.95% (107/129)	0.371
Biochemical miscarriage rate	19.24% (81/421)	18.39% (16/87)	0.854
Supernumerary Embryo	4.54 ± 3.49	3.78 ± 3.10	0.094

BMI, body mass index; FSH, follicle-stimulating hormone.

**Figure 2:**
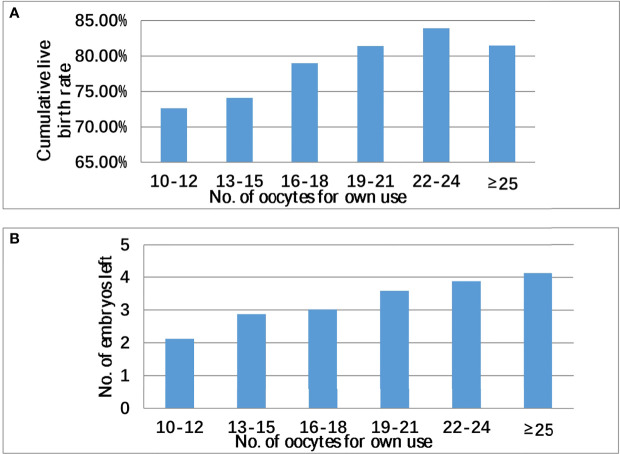
Cumulative live birth rate **(A)**, supernumerary embryo **(B)**, and number of oocytes retrieved in all patients with oocyte ≥10.

As shown in **Figure 1**, CLBR in the donation group was higher than that in the non-donation group, but this difference was not significant (82.95% vs. 81.31% p = 0.371). Oocyte donation reduced the number of supernumerary embryos because the number of supernumerary embryos in the donation group was generally smaller. As shown in **Figure 2**, in patients with ≥10 oocytes retrieved, the CLBR and the number of supernumerary embryos also become larger with the increase of number of oocytes. There was still a satisfactory CLBR (73%) when the oocyte number was 10, over two excess embryos left in those general patients.

## Discussion

This study focuses on whether current oocyte donation technology in China reduces the CLBR of donors and seeks the possibility of looking for more donors. Oocyte number is one of the positive factors affecting the CLBR (13). The more oocytes retrieved, the more embryos obtained, and patients; implantation rate increases with more opportunities to obtain implantations (14). Blyth speculated that a woman who donates eggs may reduce her own chances of pregnancy by giving away a proportion of her eggs (15). In our study, oocyte donation does not compromise CLBR among donors. Almost every reproductive center has supernumerary embryos; it is a waste of resources, and these embryos are either discarded or protected by law (16). There was also a great waste of embryos in both groups in our study, even in oocyte donation group: 3.78 embryos were left after archiving live birth. Oocyte donation reduced embryo waste and was enormously beneficial to both sharers and receivers. The assisted reproductive technology (ART) calculator has been published for predicting the minimum number of metaphase II (MII) oocytes (MII min) to obtain at least one euploid blastocyst in patients undergoing IVF/ICSI (17). This tool could also help to suggest how many embryos left to donors were appropriate; thus, the oocytes were allocated reasonably. We also hope to be able to make clear how new markers could influence the final results such as follicular fluid anti-Müllerian hormone (AMH) (18), cumulus cells (19), BMI, and AMH (20, 21), so as to provide guidance on the number of oocytes donated during egg donation, so that patients can ensure a higher probability of pregnancy and donate enough eggs.

The number of oocyte donation cycles continues to increase in regions and countries (22). For example, in the United States, the number of infertile women after age 42 utilizing autologous oocytes is very small; after age 43, autologous oocyte use in US IVF cycles is almost non-existent (23). This indicates that the age of oocyte donors is a critical factor and that there should be an upper age limit for oocyte donors: when the oocyte donors are younger, the CLBR is higher in recipients with the same age. Therefore, in women with advanced infertility, the choice of whether to accept donated oocytes was a sensible decision with respect to both clinical pregnancy success rate and economic benefit (24). It can be speculated that oocyte donation cycles will become increasingly popular around the world (25, 26). Therefore, the requirement of oocytes donated was urgent in many countries, especially in China, whose oocyte donation sources are limited.

There has been widespread concern regarding donors in China, and inquiries about donation were made after oocytes were retrieved; thus, data regarding the number of oocytes for donors do not exist (27). Moreover, the donors are free to withdraw from the oocyte donation process at any time before the recipient has had her embryo transfer. The donors and recipients did not reveal their identities to each other (28).

To the best of our knowledge, this is the first Chinese report to examine the effect of oocyte donation on donors (29). Previous studies showed that egg donation did not damage the pregnancy rate or CLBR when compared with those of standard IVF/ICSI patients (30). Some cycles even left only eight oocytes to donors while still leading to a satisfactory delivery rate (31). This may be used as a model to expand the number of eggs donated in China. What is more, beside altruism, reciprocity may be an important moral reason for people to donate gametes (32). However, these studies did not specify the embryo number. In our study, we found that IVF/ICSI patients with more than 18 oocytes (>15 oocytes for own use) annually had an average of three to four supernumerary embryos, suggesting that donating their oocytes could reduce the waste of fertility resources. These embryos could be built to establish human embryonic stem cell lines for stem cell research, and this prospect is very promising (33).

The oocyte number in the donor group was greater than that in the control group, which might be because the more oocytes one obtained, the more likely one wants to donate oocytes. There was no significant difference in age, BMI, or FSH between the two groups. The reason that the live birth rate in the donor group was higher than that in the control group might be that the invisibility conditions were better in the donor group.

Technology, ethics, and psychiatry should be taken seriously. One study interestingly found no difference between egg share donors who had been successful or unsuccessful in their own treatment regarding their feelings towards the recipient and any potential children. Moreover, there is a consensus that oocyte donors should not be paid for their contribution (34).

This was a single-center retrospective study of a limited number of IVF/ICSI cycles with oocytes meeting the donating criterion. This retrospective study has limitations, such as unavoidable shortcomings of retrospective single-center studies and the fact that some hidden influences could not be noticed. In addition, it would be better if more patients were included from multiple centers in our country.

## Conclusions

Currently, oocyte donation did not compromise CLBR, and oocyte donation can decrease the waste of embryos in patients undergoing IVF/ICSI treatment. In addition, in patients with 10 oocytes retrieved, the CLBR was still good (73%). Thus, it is possible to expand oocyte donors if the number of oocyte kept for own use was decreased from 15 to 10 after enough communication with patients.

## Data Availability Statement

The original contributions presented in the study are included in the article/supplementary material. Further inquiries can be directed to the corresponding author.

## Ethics Statement

The studies involving human participants were reviewed and approved by Institutional Review Board (IRB) of the First Affiliated Hospital of Zhengzhou University. The patients/participants provided their written informed consent to participate in this study.

## Author Contributions

JZ and ZB contributed to the conception, design, acquisition and interpretation of data, and drafting of the manuscript. YS and YZ supervised the study. All authors contributed to the article and approved the submitted version.

## Funding

This study was supported by the Natural Science Foundation of China (NO: 81801448).

## Conflict of Interest

The authors declare that the research was conducted in the absence of any commercial or financial relationships that could be construed as a potential conflict of interest.

## Publisher’s Note

All claims expressed in this article are solely those of the authors and do not necessarily represent those of their affiliated organizations, or those of the publisher, the editors and the reviewers. Any product that may be evaluated in this article, or claim that may be made by its manufacturer, is not guaranteed or endorsed by the publisher.
